# Genomics and Public Health: Development of Web-based Training Tools for Increasing Genomic Awareness

**Published:** 2005-03-15

**Authors:** Sharon LR Kardia, Jennifer Bodzin, Aaron Goldenberg, Toby Citrin, Sarah F Raup, Janice V Bach

**Affiliations:** Michigan Center for Genomics & Public Health, University of Michigan, School of Public Health, Department of Epidemiology; Michigan Center for Genomics & Public Health, University of Michigan, School of Public Health, Ann Arbor, Mich; Michigan Center for Genomics & Public Health, University of Michigan, School of Public Health, Ann Arbor, Mich; Michigan Center for Genomics & Public Health, University of Michigan, School of Public Health, Ann Arbor, Mich; Center for Genomics & Public Health, University of Washington, Seattle, Wash; Michigan Department of Community Health, Epidemiology Services Division, Genomics Program, Lansing, Mich

## Abstract

In 2001, the Centers for Disease Control and Prevention funded three Centers for Genomics and Public Health to develop training tools for increasing genomic awareness. Over the past three years, the centers, working together with the Centers for Disease Control and Prevention's Office of Genomics and Disease Prevention, have developed tools to increase awareness of the impact genomics will have on public health practice, to provide a foundation for understanding basic genomic advances, and to translate the relevance of that information to public health practitioners' own work. These training tools serve to communicate genomic advances and their potential for integration into public heath practice. This paper highlights two of these training tools: 1) *Genomics for Public Health Practitioners: The Practical Application of Genomics in Public Health Practice,* a Web-based introduction to genomics, and 2) *Six Weeks to Genomic Awareness*, an in-depth training module on public health genomics. This paper focuses on the processes and collaborative efforts by which these live presentations were developed and delivered as Web-based training sessions.

## Introduction

As genomics research continues to expand and identify potential applications for disease prevention, departments of health as well as public health practitioners will need to become increasingly aware of genomics as a public health tool. In 2001, the Centers for Disease Control and Prevention (CDC) produced a set of competencies to help integrate genomics into public health practice and training (1,2). Genomic competencies were developed for all members of the public health workforce, along with specific competencies for each functional area of public health: administration, clinical, epidemiology, health education, laboratory, and environmental health (3). These competencies are described in detail and are available from www.cdc.gov/genomics/training/competencies/comps.htm (4). Based on these competencies, a public health worker in any program at any level should be able to demonstrate a basic knowledge of the role genomics plays in disease development. This knowledge is essential for public health practitioners to integrate genomics tools into public health practice and to educate the public.

Currently, the public's understanding of genomics is derived primarily from the media, and the scientific information is often incomplete and/or inaccurate (5,6). As acknowledged in the 2002 Institute of Medicine report, *Who Will Keep the Public Healthy?*, "because few in the current public health workforce have the level of understanding of genomics that is required today, major continuing educational efforts must be undertaken to ready practicing public health professionals to use genomics effectively" (7).

During the past few years, several genomics training tools have been developed to help public health practitioners increase their awareness of the impact of genomics on public health practice, such as genetic testing for adult cancer, identifying genetically at-risk subgroups susceptible to environmental exposures, or developing new genomic technologies. These training tools aim to provide a foundation for understanding basic genomics (e.g., DNA mutations, inheritance patterns). They also help practitioners identify and translate the relevance of genomics to their own work (e.g., using family history as a genomic–environmental indicator of a person's own risk of chronic diseases). Traditionally, genomics training tools have included books, CD-ROMs, lectures, workshops, and presentations at conferences or meetings. The Genomics Toolkit is a good example of a recently developed guide designed to deliver an inventory of effective genomic tools for public health practitioners to use in program technical assistance (2,8). With improved technologies now available for developing high-tech presentations — such as synching audio, video, and PowerPoint — using the Internet to provide genomics training sessions is a logical, convenient way to increase the genomic literacy of public health professionals.

Two Web-based genomics training tools have been developed to provide genomics education to large numbers of public health practitioners nationwide. *Genomics for Public Health Practitioners: The Practical Application of Genomics in Public Health Practice* serves as an introduction, while *Six Weeks to Genomic Awareness* is a more in-depth series of six training modules. Both training tools described in this article resulted from the collaborative efforts of individuals from the CDC's Office of Genomics and Disease Prevention (OGDP), the Michigan Center for Genomics and Public Health (MCGPH), the North Carolina Center for Genomics and Public Health (NCCGPH), and the University of Washington Center for Genomics and Public Health (UWCGPH).

## Genomics for Public Health Practitioners: The Practical Application of Genomics in Public Health Practice

*Genomics for Public Health Practitioners: The Practical Application of Genomics in Public Health Practice* is a Web-based introductory presentation aimed at increasing awareness of the impact of genomics on public health and familiarizing practitioners with some basic concepts of public health genomics. Topic areas covered in this training tool include definitions (e.g., genetics, genomics, the human genome project) and applications of public health genomics (e.g., surveillance, policy, education); potential interventions based upon genomic information (e.g., modification of screening recommendations, exposures to environmental factors); challenges facing public health genomics (e.g., rapid commercialization of genetic tests, equal access to interventions); and a description of how one public health program incorporated genomics (e.g., Utah Health Family Tree Study, CDC Family History Public Health Initiative). Individuals who designed this tool developed content that would be appropriate for public health professionals with no genomics background or exposure. Conference calls, e-mails, and a Web board for posting files were used to communicate ideas among the many individuals and locations. After content was agreed upon, the module was animated and narrated for use as a Web-based presentation. An informal piloting and evaluation stage, during which public health practitioners offered feedback, provided insight on changes for the final product. Following approval by the CDC, the final presentation was launched on the Web sites of OGDP and each Center for Genomics and Public Health on August 19, 2004.

## Six Weeks to Genomic Awareness

Creating the *Six Weeks to Genomic Awareness* series was consistent with one of the main goals of the Centers for Genomics and Public Health — to develop and provide genomics training for the current and future public health workforce. An existing collaborative relationship between the Michigan Department of Community Health (MDCH) and the MCGPH afforded the capacity for developing this kind of training. The Genomics Work Group, a committee established to integrate genetics into chronic disease programs at MDCH, and whose members include representatives from the Bureau of Health Promotion and Disease Control (e.g., cancer, cardiovascular, and diabetes programs), the Bureau of Laboratories, and the Bureau of Epidemiology (including genomics and newborn screening), requested help from the MCGPH in developing a genomics training for MDCH staff. These public health professionals agreed that all program staff within their departments would benefit from introductory information on the impact of genomics on a variety of public health issues. Working with the MCGPH, the state genomics coordinator helped to identify the topic areas most relevant for state health agency employees.

During winter 2002, the plan for *Six Weeks to Genomic Awareness* began to take form. First, partners at both the MCGPH and the MDCH identified topics that would allow public health practitioners to begin integrating genomics into the functional areas of public health (administration, clinical, epidemiology, health education, laboratory, and environmental health). Twelve initial topics were identified along with detailed learning objectives, terms, and concepts, additional resources (such as Web sites), and interactive exercises. After discussions with MDCH partners about desired format and length, *Six Weeks to Genomic Awareness* was created as a six-module course to be presented onsite at MDCH in 90-minute sessions during the lunch hour through May and June 2003. Three members of the MCGPH team, two professors at the University of Michigan School of Public Health, two genetic counselors from the University of Michigan, and the state genomics coordinator at MDCH developed and presented the seminar series.

The series was designed to provide an understanding about the role of genomics throughout all public health fields, not just those programs traditionally associated with genetics (e.g., newborn screening, maternal and child health). Additionally, the course aimed to dispel myths about genetic determinism and motivate health professionals to consider the ethical, legal, and social implications of applying genomics within the public health context. The *Genomic Competencies for the Public Health Workforce *outlined by the CDC (4) were used as a backbone for session development. Modules were designed to reflect the major themes most relevant for public health — molecular genetics, genes in populations and gene–disease associations, genetic testing, gene–environment interactions, ethical, legal, and social implications, and state and national resources (Table 1).

*Six Weeks to Genomic Awareness* proved to be a successful approach to educating Michigan's public health workforce at the state level. In all, 70 program staff attended at least one session and 32 attended three or more. Attendees represented a variety of bureaus (e.g., Bureau of Laboratories), divisions (e.g., Epidemiology Services Division), and programs (e.g., Cancer, Vital Records). Following each session, evaluations were collected to gather feedback on the content, format, and effectiveness of the presentations based on a five-point rating scale (1 = poor, 5 = excellent). On average, participants rated the sessions as "very good" in terms of overall impression, usefulness of content, effectiveness of presentation, and quality of visual aids and materials. Participants also commented on the most useful part of each session and what might have been confusing or least useful and suggested additional topics to be covered in future genomics training sessions. This information was used to improve and enhance each presentation as part of the process for converting the series into the Web-based training course described below.

Given the success of *Six Weeks to Genomic Awareness* in providing genomics training to Michigan's public health workforce, MCGPH decided to convert the sessions into an online format for dissemination to public health practitioners nationwide. As work began on converting *Six Weeks to Genomic Awareness* into an online course, the Association of State and Territorial Chronic Disease Program Directors (CDD) approached the MCGPH with a request for genomics training for its members. Recognizing an ideal opportunity to improve and disseminate *Six Weeks to Genomic Awareness* to a broader audience, the CDD provided the funding necessary for ensuring the conversion of the series into a Web-based format.

Presentations given during the MDCH sessions were used as the basis for the online distance-learning modules; however, slides needed to be updated to reflect changes in the science and to include improved graphics and animations. In addition, the 90-minute format originally used was not appropriate for viewing on the Web, and the content was repackaged into 20–30-minute modules more suitable for the Web. While the evaluations collected from the MDCH onsite sessions had provided important data on the needs of public health practitioners, wider evaluation data were needed to develop the online sessions. Individuals at the CDD, CDC, NCCGPH, and UWCGPH provided expert and practical review and comment during the development of the online course.

During June and July 2004, each session was filmed and edited, and a Web template was created to synchronize the video and slide presentations. Marketing tools, such as e-mail postcards, were developed and distributed to CDD and CDC staff to announce the launch of *Six Weeks to Genomic Awareness* on July 19, 2004. A lobby page (available from www.genomicawareness.org) was developed to house all of the *Six Weeks to Genomic Awareness* training modules and to provide an avenue for individuals with questions, comments, or problems accessing the module to contact the Michigan Center for Genomics and Public Health.

The kick-off to the Web-based version of *Six Weeks to Genomic Awareness* culminated on August 19, 2004, in a one-hour live Webcast that brought together a panel consisting of *Six Weeks to Genomic Awareness* presenters, Toby Citrin, JD, and Sharon Kardia, PhD, in addition to Jean Chabut, BSN, MPH, the Chief Administrative Officer at the Michigan Department of Community Health. Public health professionals from around the country called in or submitted questions in advance for the panel to answer during the Webcast.

Between the time the modules became available online on July 19, 2004, and the end of October 2004, almost 3000 unique visitors (as determined by an individual computer's unique IP address) had visited the *Six Weeks to Genomic Awareness* Web site and had streamed more than 62,000 megabytes of content. (To put this in perspective, a data transfer of 1000 megabytes is equivalent to approximately 15 hours of viewing.)

*Six Weeks to Genomic Awareness* is also a cost-effective approach to providing genomics education. Total costs for developing the online course and producing the live Webcast were roughly $35,000. This figure does not include payment for instructor time, which was contributed. With almost 3000 individuals accessing *Six Weeks to Genomic Awareness* in just the three months tracked so far, the per-person cost of providing this genomics training is about $12. We fully expect the number of viewers to increase over time and the per-person cost of this training to decrease.

A voluntary evaluation also accompanied each of the *Six Weeks to Genomic Awareness* presentations. An analysis of evaluations (n = 41) from the first module, "Introduction to Genomics: The Human Genome" indicated that the majority of individuals viewing the session found it to be "very good" or "excellent" in terms of overall presentation (88%), usefulness of content (76%), appropriateness of Web-based format (88%), and relevance to their area of work (77%). Participants who filled out evaluation forms represented 20 states nationwide and one international location. The majority of the participants (63%) had more than 10 years of experience in their field of work, and 30% worked in public health practice, 22% in public health research, and 24% in health care provision. Other individuals worked in a range of fields from basic science, policy and legislation, nursing, geographic information systems, education, and counseling.

Additional evaluation questions asked participants to measure how knowledgeable they felt, how much new information they had learned, how interested they were in learning more, and how confident they felt in applying the information after viewing the presentation, using a 10-point scale (1 = not at all, 10 = very). Responses suggested that individuals completing the first module felt relatively more knowledgeable about the human genome than before (mean ± SD = 6.73 ± 2.07) and had learned new information about the human genome (mean ± SD = 7.48 ± 2.12). Individuals also demonstrated a great interest in learning more about the human genome (mean ± SD = 9.05 ± 1.30); however, they did not feel as confident that they would be able to apply the information to their area of work (mean ± SD = 7.20 ± 2.42). At this time, the evaluation results from the other modules continue to be compiled and analyzed.

## Collaborative Efforts

Both *Genomics for Public Health Practitioners: The Practical Application of Genomics in Public Health Practice* and *Six Weeks to Genomic Awareness* demonstrate the importance of collaboration in developing genomics training tools. Effective and appropriate education of professionals on public health genomics requires the expertise and resources of many individuals and organizations working together at all stages of development, evaluation, implementation, and dissemination.

Collaboration is vital because public health professionals nationwide are at different levels of understanding and different levels of integrating genomics into public health. Because public health genomics is a new and emerging field, it is important to encompass the needs of a wide range of audiences. Practitioners should be involved in developing training methods and materials to ensure that the final product will meet the needs of public health professionals and will relate to their work, both in terms of issues covered and language used to teach genomic concepts. Academic institutions need to share genomic research and knowledge and to provide insight into successful teaching methods, as well as to help place science in a public health context and to focus on important ethical, legal, and social issues. Technical experts, who know how to produce a distance-learning course in terms of software, equipment, and technical support, are also needed to develop valuable Web-based training. When the organizations giving and the organizations receiving the training work together, the needs of all audiences can be met more successfully.

## Future Projects

The successes of *Genomics for Public Health Practitioners: The Practical Application of Genomics in Public Health Practice* and *Six Weeks to Genomic Awareness* have motivated new projects to expand genomics training in specific disease areas. For example, at the UWCGPH, a presentation on the genetics of obesity has been developed and disseminated via the Web. *Genetics of Obesity* was offered as a live presentation at the Chronic Disease Directors Diet, Nutrition, and Physical Activity teleconference on October 14, 2004, and converted into an online audio-assisted presentation. An accompanying brochure supplements the information found in the presentation and directs public health practitioners to additional articles and Web resources. In addition, the MCGPH, the MDCH, and the Michigan Cancer Genetics Alliance are collaborating to develop a series of modules on cancer genomics for public health professionals in the MDCH Cancer Section. This process began with a needs assessment (developed with help from the NCCGPH) to target specific areas of interest. *Genomics for Public Health Practitioners: The Practical Application of Genomics in Public Health Practice, Six Weeks to Genomic Awareness*, and the cancer and obesity training sessions describe a three-tiered approach to providing genomics training: introductory, in-depth, and disease-specific. By using this approach, public health practitioners have the opportunity to understand basic concepts before being exposed to more complex topics.

Both the University of Michigan School of Nursing and School of Dentistry have used *Genomics for Public Health Practitioners: The Practical Application of Genomics in Public Health Practice* and *Six Weeks to Genomic Awareness*. Faculty from programs outside the University of Michigan have also indicated interest in linking students to the *Six Weeks to Genomic Awareness* as part of their genetic epidemiology education. One clear advantage of Web-based learning is its wide capacity for distribution; it extends easily to new and larger audiences. There appears to be a great need for genomics information by other health professionals and for training future health professionals.

## Conclusion

An introductory module, *Genomics for Public Health Practitioners: The Practical Application of Genomics in Public Health Practice*, and a more extensive training course, *Six Weeks to Genomic Awareness*, have been developed to provide quality genomics training to public health practitioners using two Web-based formats. *Genomics for Public Health Practitioners: The Practical Application of Genomics in Public Health Practice* uses computer-animated graphics with voiceover, whereas *Six Weeks to Genomic Awareness* employs live instructors in a lecture format that is taped specifically for use on the Internet. Both tools can be used to increase awareness of the impact genomics has on public health practice, to provide a foundation for understanding genomic advances, and to help translate the relevance of this information to public health practitioners' own work. These training tools demonstrate the importance of a collaborative approach, with organizations willing to share resources and expertise throughout project development and implementation. The success of both trainings also demonstrates the effectiveness of using the Web as a tool for disseminating genomics education. By developing *Genomics for Public Health Practitioners: The Practical Application of Genomics in Public Health Practice* as a Web-based introduction and converting *Six Weeks to Genomic Awareness* into an online format, more than 3000 public health professionals have been able to access genomics training via the Web. Both trainings can be accessed through links from multiple sources, including the Web sites for each of the Centers for Genomics and Public Health and the CDC's OGDP. *Genomics for Public Health Practitioners* is available from www.cdc.gov/genomics/training/GPHP/default.htm. *Six Weeks to Genomic Awareness* is available from www.genomicawareness.org.

## Figures and Tables

**Table:** Six Weeks to Genomic Awareness Session Goals

**Session 1: Introduction to Genomics: The Human Genome** The goals of this session are to provide an overview of basic molecular genetics necessary for an understanding of a genetic approach to public health and to describe how diseases are inherited at the molecular level and transmitted through families. Topics include cell structure, function, and replication; the relationship between protein, DNA, RNA, genes, and chromosomes; effects of mutations on gene and protein expression and health; Mendel’s Laws; inheritance patterns; and pedigrees.
**Session 2: Genes in Populations** The goal of this session is to introduce concepts in population genetics and genetic epidemiology and to learn how to apply this knowledge to analysis of the scientific literature and popular media. Topics include population genetics; the Hardy–Weinberg Principle; methodologies to examine the genetic basis of disease; and human genome epidemiology.
**Session 3: Genetic Testing** The goal of this session is to provide an overview of genetic testing terms, methods, and issues, including population screening. Topics include laboratory methods; test descriptions; testing limitations and complexities; informed consent process; federal and state oversight; current population-based screening programs; and nonmedical uses of genetic testing technologies.
**Session 4: Gene**–**Environment Interactions** The goal of this session is to demonstrate how a person’s genetic makeup interacts with environmental factors and what this interaction means for public health. Topics include examples of how gene–environment interactions result in disease development and the benefits and implications from research.
**Session 5: Ethical, Legal, and Social Issues** The goal of this session is to introduce the ethical, legal, and social issues arising from the application of genetic technologies in medicine and public health. Topics include ethical issues; how racial/ethnic factors affect the use, understanding, and interpretation of genetic information; policies/legislation related to genetic privacy and discrimination; gene patents; and genetic determinism.
**Session 6: An Overview of State and National Resources** The goals of this session are to provide an overview of the genetic health care delivery system, including state-specific examples; to provide an overview of the role of state and national agencies/organizations in genomics research and its application to public health practice; and to demonstrate how state and national entities might serve as resources for public health professionals seeking to maintain up-to-date knowledge about genomics and public health. Topics include types and changes to the delivery of genetic services; the genetic health care delivery system; and federal and state genomics resources.
